# Tuna Species Substitution in the Spanish Commercial Chain: A Knock-On Effect

**DOI:** 10.1371/journal.pone.0170809

**Published:** 2017-01-26

**Authors:** Ana Gordoa, Gustavo Carreras, Nuria Sanz, Jordi Viñas

**Affiliations:** 1 Department of Marine Ecology. Centro de Estudios Avanzados de Blanes, Spanish National Research Council (CSIC). Blanes, Girona, Spain; 2 Laboratory of Genetic Ichthyology. Department of Biology, Faculty of Sciences, University of Girona, Spain; Universita degli Studi di Bari Aldo Moro, ITALY

## Abstract

Intentional mislabelling of seafood is a widespread problem, particularly with high-value species like tuna. In this study we examine tuna mislabelling, deliberate species substitution, types of substitution and its impact on prices. The survey covered the commercial chain, from Merca-Barna to fishmongers and restaurants in the Spanish Autonomous Community of Catalonia. To understand the geographic extent of the problem we also sampled Merca-Madrid, Europe’s biggest fish market, and Merca-Málaga for its proximity to the bluefin tuna migratory route and trap fishery. Monthly surveys were carried out over one year. The results showed a high deficiency in labelling: 75% of points of sale and 83% of restaurants did not specify the species, and in those cases the name of the species had to be asked. A total of 375 samples were analysed genetically, the largest dataset gathered in Europe so far. The identified species were *Thunnus albacares*, *Thunnus thynnus* and *Thunnus obesus*. Species substitution began at suppliers, with 40% of observed cases, increasing to 58% at fishmongers and 62% at restaurants. The substitution was mainly on bluefin tuna (*T*. *thynnus*), 73% of cases. At restaurants, only during the bluefin fishing season, we observed a decrease of Bluefin tuna substitution and an increase of reverse substitution revealing some illegal fishing. The effect of species substitution on species prices was relevant: *T*. *obesus* increased its price by around €12 kg^-1^ when it was sold as bluefin. In view of the deficiency of labelling, the abuse of generic names and the lack of the bluefin catch document, we conclude that the Spanish regulations are ineffective, highlighting the need for policy execution, and the urgent need for information campaigns to Spanish consumers.

## Introduction

Mislabelling of seafood products has been documented worldwide over decades, becoming a significant concern in domestic and international markets [1]. Mislabelling can be accidental and is difficult to prevent due to the difficulty of distinguishing between species of similar morphological features: when they are caught simultaneously, accidental mislabelling occurs on board [2–6]. Once mislabelled on board or at landing, the error persists along the entire food chain to the consumer. However, intentional mislabelling or fraud has been widely detected for economic gain or to meet market demand by mislabelling low-market value fish as high-value species [7, 8] and has been found in a wide range of seafood [7, 9–17]. Either way, both accidental mislabelling or intentional species substitution impact on a wide variety of fields, concealing illegal exploitation which could affect estimates of stock sizes or overfishing [18, 19] or facilitating the traffic of protected species [20] and also increasing risks for public health [21–23]. Moreover, species substitution increases retailers’ profit to the detriment of consumers [24] and also of legal producers and retailers [13] and restaurants [7]. In addition, the present-day globalization of the seafood trade and processed products facilitates fraudulent market substitution [24].

Tuna is one of the world’s most traded, valued and sought-after fish species. The global tuna market is worth in the region of $6 billion, with annual catch volumes of around 4 million tonnes, and accounts for around 10% of the world’s international seafood trade [25]. There are various related members of the tuna family with different market values: in the Japanese market bluefin tunas are the most highly valued [26], but in limited volumes, representing only 3% of the Japanese market share [27]. This is understandable according to the FAO’s catch statistics, in which the bluefin species, Pacific and Atlantic together, represent barely 2% of the total tuna landing catch, which is dominated by skipjack and followed by yellowfin and bigeye [28]. Tuna species, because of their large size, are sold in portions or fillets which are hard to identify since their distinguishing morphological features are no longer detectable. This raises the possibility of intentional substitution in the commercial tuna market: substitution of bluefin with other tuna which are less valuable and also more abundant on the market. Tuna species substitution is commonly observed [29, 30] but not limited by mislabelling within species: DNA barcoding revealed that “white tuna” sushi (*Thunnus alalunga*) were found to be “tilapia” (*Oreochromis mossambicus*) [17]. Tuna mislabelling in international trade might have different sources, and among them we have to consider the role of China, the world’s largest producer, consumer and exporter of seafood [31]. Tuna is one of the main unprocessed fish species imported by this country, but part of it is processed and re-exported [32]: however, China lacks specific provisions for labelling [8]. On the other hand, Spain is one of the main importing countries of fresh and frozen tuna but also one of the leading exporters [33] and one of the main harvesting countries. Approximately 20% of the eastern stock of Atlantic bluefin tuna is caught by Spanish fisheries, mostly traps and purse seiners, in fishing grounds close to its coast [34–36]. The presence of bluefin tuna along the Spanish coast along their spawning migratory route [37], the deep social rootedness due to centuries of use of traditional tuna traps, and most recently the fattening facilities, also used as tourism activities, generates the false popular perception that “atún” (tuna) is only represented by “atún rojo” (bluefin). However, the reality is different, the bluefin catch of the Spanish fleet does not reach 1% of the total tuna catch [38] similar to the contribution of this species to the global tuna market [39]. European policies regulating seafood labelling (EUR-Lex 2014) [40] protect the interests of consumers by providing the information concerning the commercial designation of the species, responsibility of the Members states, and for the main commercial species it is clearly based on the principle of “one species-one name.” The Spanish commercial names for tuna are specific at the state level, where the generic name of “atún” (tuna) is only permitted for some tuna species that are hardly ever found in the Spanish market (S1 Table). However, there is inconsistency and contradiction between the accepted state and regional names. The labelling regulations accept that in some Autonomous Communities the generic name “atún” can also be used for bluefin tuna.

Given the background presented above–tuna trade market, high prices and differences between species, the lower catch of the most valuable ones and the impossibility of distinction, as they are sold in cuts, and weak or ambiguous legislation, added to local consumer lack of information show the need for a more in-depth look at the tuna market.

The introduction of molecular identification methods has helped to identify seafood mislabelling. The DNA barcoding process has proven successful in identifying fish species [41–44], and has been applied for numerous seafood products [13, 45], including raw or cooked specimens [17, 21]. However, DNA barcoding suffers some drawbacks for tuna species identification, particularly in the discrimination between the closely related Neothunnus species (*T*. *albacares*, *T*. *tonggol* and *T*. *atlanticus*) [46]. Alternatively, the combination of two molecular markers–a fragment of the high variable mitochondrial control region (mtDNA CR) together with the ITS nuclear segment–has proven to be extremely useful in the identification of all the tuna species, even among the Neothunnus tribe, and also in the case where introgression of mitochondrial DNA among tuna is present [46].

In this study we used this genetic methodology to investigate the incidence of mislabelling and substitution of species concerning the genus *Thunnus*. To understand where mislabelling occurs within the seafood production chain, this study covers the commercial chain from wholesalers (Barcelona Central Market) to the final seafood retailer (fishmongers and restaurants) in the Spanish Autonomous Community of Catalonia. The study was undertaken over a 12-month period to investigate whether the strong seasonality of the local bluefin fishery plays any role in fraudulent commercial tuna substitution. In addition, and in order to understand the geographic extent of the problem, we also included two additional Central Markets (wholesalers). Merca-Madrid, Europe’s largest fish market, and Merca-Malaga, which was selected for its close proximity to the bluefin tuna fishing ground and trap fishery. Moreover, in none of these regions can the generic name “atún” be legally used for bluefin.

## Material and Methods

### Sample Collection

The sampling process covered the various stages of the commercial chain, from wholesalers to restaurants. Samples were collected from tuna for sale at these locations, regardless of the label (specific, generic or none). The tuna found at points of sale (wholesalers and fish shops) were fresh or unfrozen. Fish samples were collected monthly over a twelve-month period (November 2014-October 2015) in towns along the entire Catalan coast and in the capitals of the coastal provinces (Girona, Barcelona and Tarragona) (S1 File). The sampling plan considered the collection of 36 samples for each quarter-year from fish shops (fishmongers and large stores) and an equal number from restaurants, both evenly distributed between quarter-years and provinces. Similarly for wholesalers, the plan for the three central markets (Málaga, Madrid, Barcelona) considers the same number of samples: 36 samples per quarter-year and market. Since sampling began in November the quarters did not correspond to the calendar year (Q1 = November-January; Q2: February-April; Q3 = May- July; Q4 = August-October). We presumed that large stores operate under the guidelines of their corporations and may well behave differently from fishmongers. Consequently, we show their respective results separately.

Samplers were instructed to simulate being fish buyers or restaurant clients. Samplers were also trained to gather specific information from labels and menus, and when the information was weak or incomplete they had to ask, casually, about the species offered to the fish seller or waitress and when they did not know they asked the owner or cook. For each fish sample of tissue collected in fish shops (fishmongers and large stores) or wholesalers, the following information was recorded: date, town, shop/market name, species’ commercial name, scientific name and price (€/kg). Similarly, the information recorded for restaurants was date, town, restaurant´s name, commercial name on the menu, type of dish (raw or cooked) and its price. In those cases where samplers read (on the label or menu) or were told that the fish was “atún rojo” (bluefin”), later, at the time of paying, they asked if the restaurant had the DCA (“Bluefin Catch Document”) and knew of its existence. The DCA is a document providing complete traceability of bluefin: this document is only mandatory for bluefin and has to trace the fish’s commercial route all the way through to the end consumer.

Each sample, fresh or cooked, was coded and a block of tissue was taken placed in tubes filled with 96% ethanol. The code and the corresponding information of each sample were immediately recorded in a SharePoint with all the additional information (observations, comments and images). Samples were genetically analysed for species identification and these results were compared with the species reported at sampling.

### Species Identification

The methodology used for genetic identification was anonymous sample labelling and with the presence of random positive controls distributed among the samples to ensure that there was no bias or error during the procedure. The laboratory procedure was based on that described in Viñas and Tudela [46]. Briefly, when the tissue arrived at the Laboratory of Genetic, it was processed by DNA extraction using REAL pure genomic DNA Extraction Kit (Durviz, Valencia, Spain). Subsequently, the mtDNA control region was amplified by PCR and sequenced as described in the above mentioned study. Finally, sequence characterization and species identification was carried out by two procedures: the mtDNA CR sequences obtained were compared using FINS (Forensically Informative Nucleotide Sequencing) [47], a methodology using the sequence database of the study by Viñas and Tudela [46]. In addition, the sequences were also compared to the Genbank data base by BLAST [48] using the high similarity algorithm. In the case of possible detection of introgressed mitochondrial DNA, mainly when *Thunnus alalunga* were detected, to differentiate between introgression and actual species identification, these samples were resequenced by the second alternative nuclear marker ITS [46, 49]. Similarly to the procedure of mtDNA CR, ITS sequences were analysed using FINS and BLAST.

## Results

Finally, 86% of the planned sampling size was reached (Table 1). A total of 375 biological samples were finally collected (S2 File and S3 File), representing the largest dataset collected to date in Europe. Among these samples, 130 were collected from restaurants, 111 from fish shops, and 134 from Central Markets.

**Table 1:** Number of samples collected per quarter in fish shops and restaurants in each Catalan province and the number of samples collected per quarter at Central Markets.

	Girona	Barcelona	Tarragona	Madrid	Málaga
**Restaurants**	12/10/10/12	12/11/9/9	12/12/11/10		
**Fish shops**	10/13/9/9	13/10/7/5	7/10/10/8		
**(fishmongers & large stores)**					
**Central Markets**		12/12/12/12		12/12/2/12	12/12/12/12

Genetic analysis allowed the identification at the species level of 100% of the samples. Fourteen (3.7%) of the specimens were detected as introgressed DNA and subsequently resequenced with the alternative nuclear marker.

### Traded Species

The results of the genetic analysis (S2 Table) showed that only three species of tuna were traded in points of sale (wholesalers and fish shops): yellowfin (*Thunnus albacares*) representing 45% of the total number of samples analysed, followed by bluefin tuna (*Thunnus thynnus*) with 36% and bigeye (*Thunnus obesus*) around 19%. Similar results were found at restaurants: yellowfin (50%), followed by bluefin (34.5%) and bigeye (17%), but albacore (*Thunnus alalunga*) (0.7%) and Atlantic bonito (*Sarda sarda*) (0.7%) were also detected.

**Fig 1:**
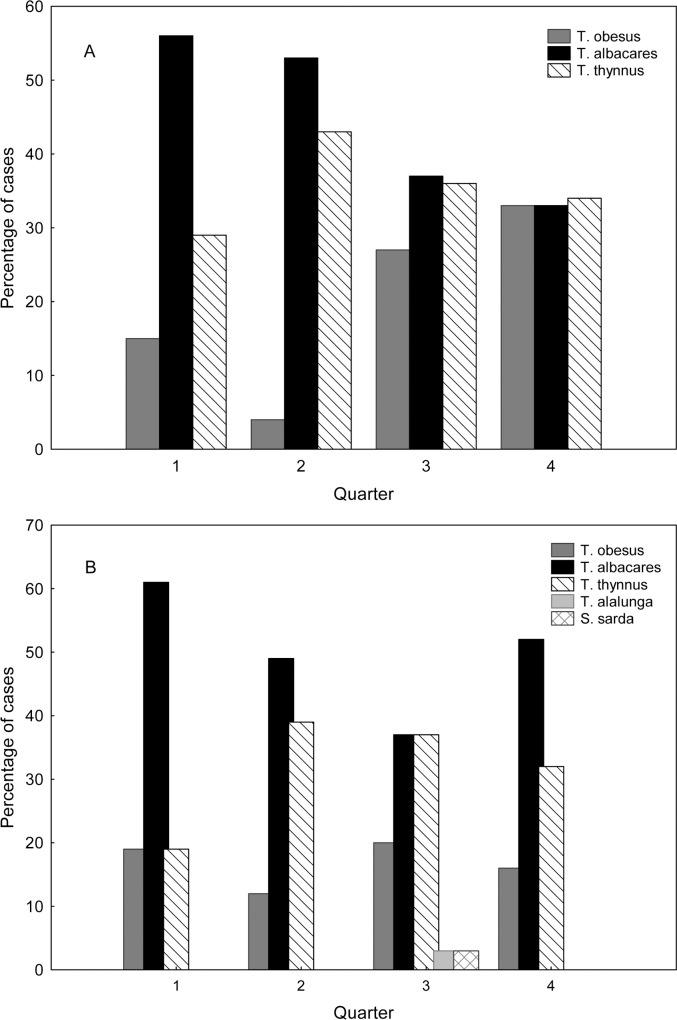
Percentage of each species identified by genetic analysis found per quarter. At points of sale (A) and at restaurants (B).

The traded species displayed seasonal variability (Fig 1). At points of sale, yellowfin decreased from the first to the last quarter while bigeye showed a reverse trend. In the last quarter the three species figured evenly in the market. At restaurants, bigeye tuna did not show any trend and its contribution was always below 20%. Yellowfin was always the most common species, but during the third quarter (May-July) bluefin reached the same level, coinciding with the bluefin fishing season in the Mediterranean region.

### Labelling

From a total of 245 points of sale, in 185 cases the labelling did not specify the species with either scientific or commercial names. Consequently, the samplers had to ask the species name at 75% of points, but in 9% of them they did not receive any answer, as sellers did not know (or “did not answer”) the name of the species, which finally had to be labelled as “undefined.” The specifications in the restaurants’ menus were even lower: 107 out of 130 restaurants had to be questioned about the tuna species offered on the menu, and 17% of them gave no specific answer and so the species were also classified as “undefined.”

The results showed some labelling differences between point-of-sale categories (Table 2), wholesalers and fish shops. Moreover, differences within fish shops were also observed. Fishmongers rarely used specific names, either scientific or commercial, while large stores mostly labelled with scientific names. The generic term “tuna” is the most common name used in every case, but uninformative at species level.

**Table 2:** Percentage of each type of name used in labels at different stages of the commercial chain.

	Scientific & Commercial	Scientific only	Commercial only	Generic only	None
**Central Markets**	**14.9**	**6.7**	**11.9**	**19.4**	**46.2**
**Large stores**	**13**	**52.2**	**4.3**	**21.7**	**8.7**
**Fishmongers**	**1.1**	**4.5**	**1.1**	**38.6**	**54.5**
**Restaurants**	**0**	**0**	**18**	**82**	**0**

The Bluefin Catch Document (DCA) was not found in any of the sampled points of sale and only in 4 of the 130 restaurants. Moreover, the apparent lack of awareness of the DCA was high, 36% of the points of sale and 66% of the restaurants declaring they did not know it.

### Commercial Species Substitution

**Fig 2:**
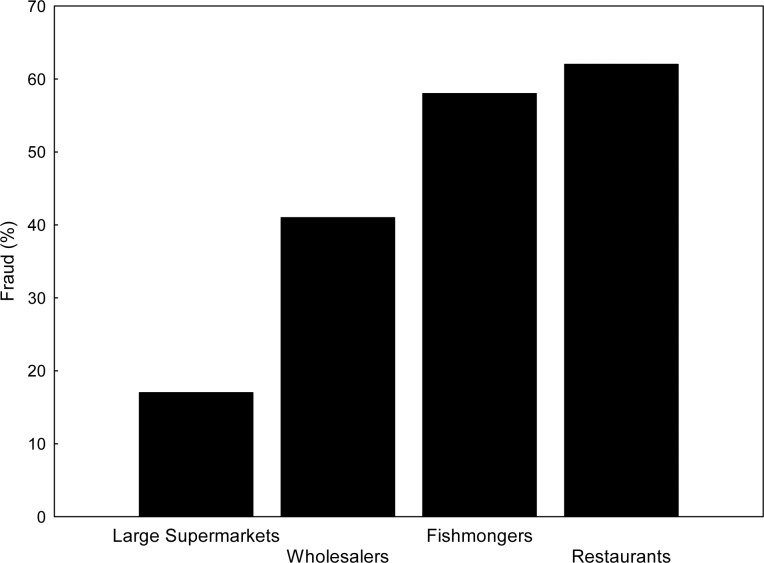
Percentage of total species substitution along the commercial chain.

The result of the comparison of species identified by genetic analysis and the species name recorded on the labels or after asking reveals a species substitution of 37% in points of sale and 48% at restaurants. The “undefined” samples (unknown or undeclared species name) also mislead consumers, and when these cases are included the global volume of substitution increases by up to 44% in points of sales and 62% at restaurants. This deception is cumulative along the commercial chain, beginning at suppliers, with 40% of observed cases, increasing to 58% at fishmongers and 62% at restaurants (Fig 2). The results of large stores must be considered with caution due to the small sample size, which was smaller than that considered in the sampling design because of the scarcity of tuna sales in this type of fish shops. In any case, large stores also showed better labelling so we cannot discard the possibility that their suppliers and/or commercial standards are stricter regarding traceability. Species substitution varies between quarter-years but no clear seasonality was observed: it decreased in fishmongers and restaurants during the second quarter, and in restaurants it remained practically unchanged during the third quarter (Table 3), with a similar trend observed in wholesalers.

**Table 3:** Percentage of global species substitution per quarter at different stages of the commercial chain. (n = number of samples).

	Q1 (Nov.-Jan.)	Q2 (Feb.-Apr.)	Q3 (May.-Jul.)	Q4 (Aug.-Oct.)
**Central Markets (n = 134)**	45	23	54	48
**Large stores (n = 23)**	0	0	0	100
**Fishmongers (n = 88)**	73	40	62	62
**Restaurants (n = 130)**	70	51	53	74

**Fig 3:**
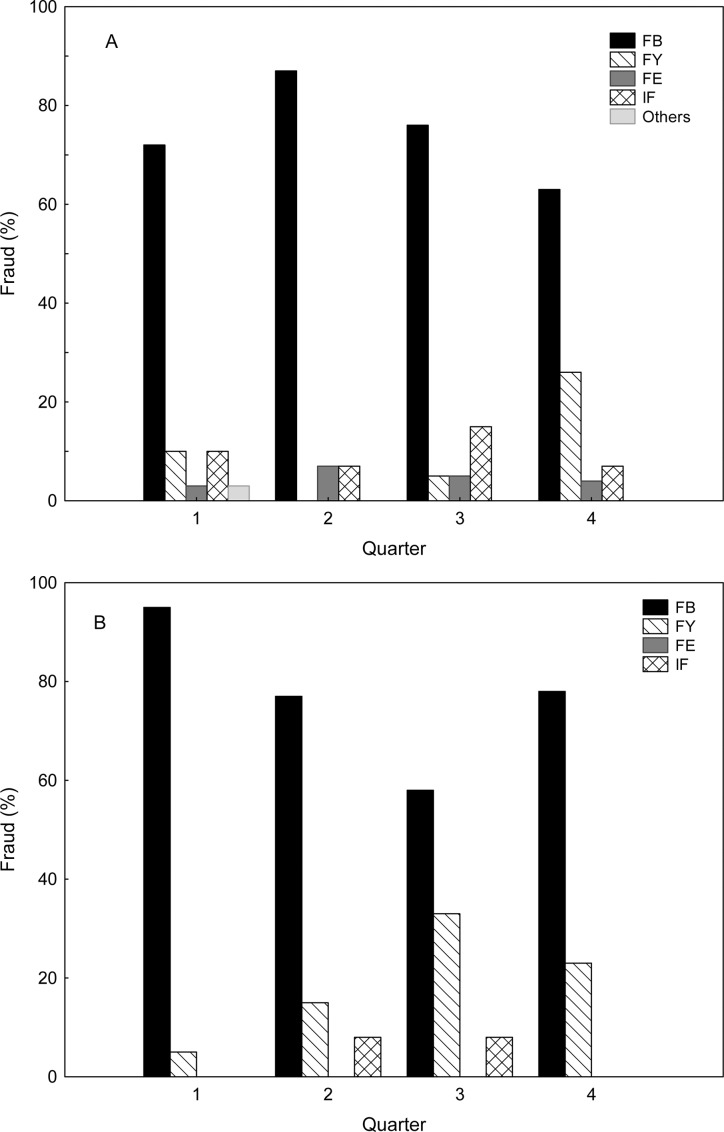
Percentages of types of fraud per quarter. At points of sale (A) and at restaurants (B). FB: declared bluefin and turned out false, similarly FY and FE when Yellowfin and Bigeye were declared respectively. IF corresponds to the percentage of cases where the name was not declared.

The results clearly showed that the species substitution was mostly on bluefin tuna. There was no indication of differences between restaurants and points of sale. In both cases, 73% of the observed substitutions, fish labelled or declared as bluefin tuna turned out to be false. The type of substitution and its seasonal variability (Fig 3) showed that at points of sale deception on bluefin tuna decreased during the fourth quarter, accompanied by an increase on yellowfin, coinciding with the above-mentioned increase of bigeye frequency in our samples. Furthermore, points of sale showed an increase of reverse species substitution during the third quarter, coinciding with the bluefin fishing season, while at restaurants, fraud on bluefin fell to its minimum value during the third quarter.

Merca-Málaga results were unexpected: despite its closeness to the fishing Spanish traps, bluefin tuna was rarely commercialised in this market. From a total of 48 samples, only 3 were identified as bluefin tuna and all were labelled as Bigeye tuna, a reverse substitution indicative of illegal origin.

### Species substitution Impact on Commercial Prices

The analysis of prices at point of sales (wholesalers and fish shops) contributes to an understanding of the direction of species substitution. The results showed differences between the commercial price of each species when sold with and without mislabelling (Table 4), demonstrating the commercial profit of planned deception. Bigeye, correctly labelled, had an average price of €8.8 kg^-1^: when sold as yellowfin, its average price increased by €2 and by €12 if it was sold as bluefin. Bigeye prices also increased when the species name was not reported. Yellowfin, with an average price of €12.8 kg^-1^, doubled its market price when it was sold as bluefin and its price increased by around €8 kg^-1^ when the species name was unstated. Bluefin tuna presented the highest market price, around €25.6 kg^-1^, but when it was sold under another species name (reverse fraud = high-market value fish as low-value species), the result was a depreciation of its commercial value. In the following section we discuss whether this depreciation should be interpreted as a loss of profit.

**Table 4:** Average price (€ kg^-1^) of tuna species under different commercial names. [%95 CI] estimated from the samples collected at points of sales.

Identified species	Species indicated in the labels			
	Bigeye	Yellowfin	Atlantic Bluefin	Undefined
**Bigeye**	8.8 [5.5–12.2]	10.3 [7.7–12.9]	20.4 [17.6–23.2]	18.9 [-6.7–6.4]
	n = 6	n = 11	n = 26	n = 2
**Yellowfin**	13.1 [2.1–24]	12.8 [10.4–13.9]	23.1 [21.4–24.7]	20.1 [15.5–25.1]
	n = 4	n = 56	n = 41	n = 10
**Atlantic Bluefin**	19.6 [14.1–25.2]	15.4 [-0.7–31.4]	25.6 [24.1–27.1]	17.5 [7.1–27.9]
	n = 6	n = 3	n = 73	n = 6

## Discussion

The result of this study revealed non-compliance with the Spanish labelling regulations and also with the DCA which is mandatory for bluefin tuna. Moreover, this study showed the high level of tuna species substitution along the commercial chain and its impact on the commercial price of tuna species.

The genetic analysis revealed that yellowfin is the most common species found in this study. However, at points of sale its contribution declined in summer and autumn while bigeye incidence increased. The rise of bigeye could be just a temporary change or indicative of an actual change in the tendency of the Spanish tuna market. Yellowfin was also the species most commonly found at restaurants, with a decrease only observed during the third quarter and recovering immediately afterwards. At restaurants, the presence of bigeye tuna was low and does not follow the increasing trend observed at points of sale, so we presume that most bigeye tuna is sold directly to the public.

The European labelling regulations establish compulsory information such as the scientific name and the corresponding commercial denomination according to the official list of names of each member state (EU Regulations n° 1379/2013), generally based on the one species-one name principle. The Spanish list of commercial names for tuna species (S1 Table) is specific for those tuna species that are most common in the Spanish market (*T*. *thynnus*, *T*. *albacares*, *T*. *obesus*, *T*. *alalunga*). The generic and vernacular name “atún” can only be used for species that are rarely commercialized in Spain, but there are exceptions because the regional vernacular names are also accepted. This is the case for bluefin in Catalonia, our case study, which can be labelled as “tonyina” which is also the Catalan translation of the term “atún,” generic and unspecific, and the one species-one name principle is lost. This scenario, and the consumers’ perception, either at national or regional level, that “atún” or “tonyina” is always “atún rojo” (bluefin), explains why mislabeling goes unnoticed by the public. The degree of unlabelled or unspecific cases, similar to the findings of other studies on tuna [50], was observed from the beginning of the commercial chain, starting with 66% at wholesalers. These results suggested that labelling is overlooked by control inspections.

The results showed that the use of the unspecific generic name hamper the detection of species substitution. This is also covered by the attitude of consumers, who rarely ask about the tuna species at the time of buying, because in Spain the public are usually unaware of the existence of other tuna species and are not well informed about fish products and policy. In this scenario of incomplete information and consumer ignorance, deception conditions are favourable [51], as was proved in this study.

We observed that species substitution was cumulative along the commercial chain; the problem begins at suppliers, with 40% of species substitution in the wholesale markets, increasing to 58% at fishmongers and reaching a similar level in restaurants (62%), but lower than what was recently reported for Bluefin tuna (95%). by Oceana in restaurants of Brussels [52] The results of our study revealed that fishmongers are greatly deceived by wholesalers but they also deceive the next level, consumers and restaurants. The different typologies of species substitution showed that the predominant practice was on bluefin, which exhibits differences between different levels of the commercial chain. At restaurants, substitution of bluefin decreased during the third quarter, reaching levels below those observed at points of sale. The third quarter matches the bluefin tuna fishing season of Spanish fisheries, and we hypothesize that during this period restaurants are offering more bluefin tuna but buying it directly from illegal catch suppliers. Thus, this reduction of substitution, which might be interpreted as a positive signal for consumers, would be negative for legal bluefin tuna providers. The observed deception which targeted bluefin tuna is consistent with worldwide findings, where species of higher value are replaced with species of lower value [12, 17, 53]. However, we also found reverse deception—commercialization of bluefin tuna labelled as a cheaper species–which is a way of facilitating the commercialization of illegally caught bluefin. Reverse deception was detected in each season but increased during the bluefin fishing season. We hypothesize that illegal catch during the tuna fishing season reduced substitution of bluefin tuna in restaurants and increased reverse deception at points of sale. However, it should be made clear that the assessment of illegal fishing is beyond the goal of this project and is currently unapproachable. Particular attention should be paid, in this context, to the total non-compliance by fish sellers with the required bluefin documentation (DCA) which identifies the origin of the catch and guarantees its traceability.

The profit from species substitution, lower-priced species marketed commercially as higher-priced species, has been detected in many different groups of species [10, 12, 18, 54]. In this study, the economic profit of this practice was clearly demonstrated by the price differences of less valuable tuna when sold with or without species substitution. In particular, bigeye tuna multiplied its market price more than twice when it was sold as bluefin. The negative impact of species substitution would increase if the increase observed in the commercialization of bigeye tuna in the Spanish market is confirmed. There is also a noteworthy behaviour of Spanish market prices: bigeye and bluefin are the most valuable tuna in the Japanese sashimi market and both command high prices [55]. The low price of bigeye in the Spanish market is hard to understand unless it represents a low-value segment of the bigeye catches, smaller individuals with low fat and texture. Reverse deception has a direct negative impact on bluefin tuna prices, with a minimum depreciation of €10 kg^-1^. Accurate determination of the total financial loss at both extremes, to the consumer and to legal producers, is not simple but it does appear to be relevant.

Although the present regulations on Bluefin tuna traceability documentation (DCA) should prevent deception, our findings showed a high degree of non-compliance, a total default at points of sale. Moreover, a high percentage of the respondents declared that they did not know the existence of the DCA because it was never requested by inspectors. In addition, the present regulations on tuna labelling are inconsistent and violate the one species-one name principle [40]. Although bluefin tuna has a specific name at the state level, in five Autonomous Communities the generic name is also accepted, and this generic name is shared by five different species of tuna. Consequently, the present labelling regulations favour the uncertainty of labelling. The harmful consequences of mislabelling go beyond economic consequences and may result in health hazards [22]. The authenticity of food is one of the aspects required to assess food security and to ensure fair competition between producers and processors [56].

Consumers are becoming more demanding about what they eat, but their efforts are undermined by the lack of knowledge and the inability to discriminate between species. How can consumers differentiate between tuna species when most of the time they are eating a different species from what they think? In our survey, 97% of tuna sold at fishmongers and around 82% of the menu listings gave no indication of what species was being served, very similar figures to those found in a previous study carried out in the USA [50]. When the fish seller or restaurant staffs were asked, on average more than 60% of the species descriptions were incorrect. Consequently, potential differences in consumers’ appreciation of taste or excellence would be attributed to differences between cooks or differences in fish preservation. Since substitutability between tuna fish species depends upon the consumers’ perception [55], they require clear and accurate information to make choices about the food they buy, so the food supplied must be exactly what the label says [26].

The results of this study are based on sound data, the largest dataset gathered in Europe so far, but they should not be extended automatically to other areas. In summary, the high level of substitution of bluefin tuna, from wholesalers to restaurants, creates the incorrect impression that this is the most widely traded tuna species in the Spanish market. Fair trading is not a guarantee: consumers, restaurants and producers incur economic losses, as do a high percentage of fishmongers. Consumers are misinformed about tuna species sold which makes them unable to differentiate between species and has a negative financial impact on them. We conclude that the Spanish regulations on labelling and on bluefin catch certificates have proven to be ineffective, highlighting the need for policy enforcements and a review of the accepted commercial names. Several actions should be taken, efforts should be made to have more effective control on labelling and labelling authenticity, including genetic testing, and on the required documentation, and it needs to be accompanied by dissuasive sanctions, proportionate to deception turnover and its associated risks and economic losses. There is also an urgent need for information campaigns addressed to Spanish consumers: species substitution must be fought from above with efficient control and from below through well-informed and conscientious consumers.

## Supporting Information

S1 Table Tuna species name (commercial designations) accepted in Spain and in littoral Autonomous Communities. (DOCX)

S1 File Primary data: codes of sampled areas. (PDF)

S2 File Primary data: point of sales. (PDF)

S3 File Primary data: restaurants. (PDF)

S2 Table FASTA sequences. (XLSX)
