# Supplementary material for: Tuna Species Substitution in the Spanish Commercial Chain: A Knock-On Effect
Source: PLoS One. 2017 Jan 26;12(1):e0170809. doi: 10.1371/journal.pone.0170809 (PMC5268641; doi:10.1371/journal.pone.0170809)
Supplement: S1 Table — (DOCX) [file pone.0170809.s001.docx]

**S1 Table. Tuna species name (*commercial* designations) accepted in Spain and in littoral Autonomous Communities.**

|  | Spain | Andalucía | Asturias | Baleares | Canarias | Cantabria | Catalonia | Valencia | Galicia | Murcia | País Vasco |
| --- | --- | --- | --- | --- | --- | --- | --- | --- | --- | --- | --- |
| *T. alalunga* | Atún blanco/ Bonito del norte/ Albacora | Atún blanco/ Bonito  del norte | Mono | Bacora/ Ullada | Barrilote | Bonito del norte | Bacora | Bacora | Bonoto do Norte |  | Hegaluze |
| *T. obesus* | Patudo /  Atún de ojo grande | Patudo | Bonita/  Obeso |  | Tuna |  |  |  | Patudo |  | Begihandia  Moja |
| *T. albacares* | Rabíl /  Atún de aleta amarilla |  |  | Tonyina groga | Rabíl |  |  |  | Atún amarelo |  | Errolflin |
| *T. thynnus* | Atún rojo/  Atún de aleta azul | Atún rojo | Atún/  Bonita/  Zurdo | Tonyina | Atún rojo/  Patudo^1^ | Albacora/  Cimarrón | Tonyina | Tonyina | Atún vermello | Atún | Hegalabur |
| *T. tonggol* | Atún tongol/  Atún |  |  |  |  |  |  |  | Atún do Índico |  |  |
| *T. atlanticus* | Atún de aleta negra/ Atún |  |  |  |  |  |  |  |  |  |  |
| *T. orientalis* | Atún del Pacifico/ Atún |  |  |  |  |  |  |  |  |  |  |
| *T. maccoyii* | Atún del sur / Atún |  |  |  |  |  |  |  |  |  |  |
| *Allothunnus*  *fallai* | Atún lanzón/  Atún |  |  |  |  |  |  |  |  |  |  |
