# Supplementary material for: Tuna Species Substitution in the Spanish Commercial Chain: A Knock-On Effect
Source: PLoS One. 2017 Jan 26;12(1):e0170809. doi: 10.1371/journal.pone.0170809 (PMC5268641; doi:10.1371/journal.pone.0170809)
Supplement: S1 File — (PDF) [file pone.0170809.s002.pdf]

Province	Towns	Codes
Barcelona	Barcelona City	B0 and B4
Tarragona	Tarragona City	T0 and T4
Girona	Girona City	G0 and G4
Girona	llança	G1
Girona	Port de la selva	G1
Girona	roses	G1
Girona	empuriabrava	G1
Girona	s. pere pescador	G1
Girona	léscala	G1
Girona	estartit	G1
Girona	begur	G2
Girona	palafurgell	G2
Girona	palamós	G2
Girona	S. antoni calonge	G2
Girona	Platja de aro	G2
Girona	sagaró	G3
Girona	S. feliu de guixols	G3
Girona	tossa de mar	G3
Girona	lloret	G3
Girona	blanes	G3
Barcelona	malgrat de mar	B1
Barcelona	calella	B1
Barcelona	arenys de mar	B1
Barcelona	caldes de estrac	B1
Barcelona	s. andreu de llavaneres	B1
Barcelona	mataró	B2
Barcelona	vilassar demar	B2
Barcelona	premia de mar	B2
Barcelona	el masnou	B2
Barcelona	montgat	B2
Barcelona	casteldefels	B3
Barcelona	sitges	B3
Barcelona	vilanova i la geltrú	B3
Barcelona	cubelles	B3
Tarragona	cunit	T1
Tarragona	segur de calafell	T1
Tarragona	calafell	T1
Tarragona	roda de bará	T1
Tarragona	s. salvador	T1
Tarragona	creixell	T1
Tarragona	toredembarra	T1
Tarragona	altafulla	T1
Tarragona	tamarit	T1
Tarragona	vila-seca	T2
Tarragona	salou	T2
Tarragona	cambrils	T2
Tarragona	miami platja	T2
Tarragona	hospitalet del infant	T2
Tarragona	calafat	T2
Tarragona	ametlla de mar	T3

Tarragona	perelló mar	T3
Tarragona	cap roig	T3
Tarragona	ampolla	T3
Tarragona	delta del ebre	T3
Tarragona	s. carles de la rapita	T3
Tarragona	alcanar	T3
