# Supplementary material for: Tuna Species Substitution in the Spanish Commercial Chain: A Knock-On Effect
Source: PLoS One. 2017 Jan 26;12(1):e0170809. doi: 10.1371/journal.pone.0170809 (PMC5268641; doi:10.1371/journal.pone.0170809)
Supplement: S2 File — (PDF) [file pone.0170809.s003.pdf]

muestra	MesAño	Q	Provincia	Zona	DCA	Código	DCA	Scientific name	Commercial name	€/kg	Pregunta tipo atún	Resultado	declarado
Gran superficie	6/2015	3	Barcelona	B0	No aplica	G0615BCR	No aplica	unspecified	unspecified	12,5	Aleta Amarilla	Thunnus albacares	Thunnus albacares
Gran superficie	6/2015	3	Tarragona	T0	No aplica	G0615TCR	No aplica	Thunnus alabacares	Atún de aleta amarilla	15	Atún de aleta amaril	Thunnus albacares	Thunnus albacares
Gran superficie	6/2015	3	Girona	G0	No aplica	G0615GCR	No aplica	unspecified	unspecified	15	Aleta amarilla	Thunnus albacares	Thunnus albacares
Gran superficie	12/2014	1	Girona	G0	No aplica	G1214GCR	No aplica	Thunnus obesus	Tonyina	15		Thunnus obesus	Thunnus obesus
Pescadería	6/2015	3	Tarragona	T4	No aplica	P0615T40	No aplica	unspecified	unspecified	36	- sin especificar -	Thunnus thynnus	- sin especificar -
Mercas	7/2015	3	Málaga	MML	No aplica	M0715ML2	No aplica	Thunnus alabacares	Atún de aleta amarilla	4	Thunnus alabacares	Thunnus albacares	Thunnus albacares
Mercas	12/2014	1	Málaga	MML	No aplica	M1214ML4	No aplica	Thunnus obesus	Atún de ojo grande	6		Thunnus obesus	Thunnus obesus
Mercas	8/2015	4	Málaga	MML	No aplica	M0815ML1	No aplica	unspecified	unspecified	7	Aleta Amarilla	Thunnus obesus	Thunnus albacares
Mercas	5/2015	3	Málaga	MML	No aplica	M0515ML2	No aplica	Thunnus alabacares	Atún de aleta amarilla	7	Thunnus alabacares	Thunnus albacares	Thunnus albacares
Mercas	7/2015	3	Málaga	MML	No aplica	M0715ML1	No aplica	Thunnus alabacares	Atún de aleta amarilla	7	Thunnus alabacares	Thunnus albacares	Thunnus albacares
Mercas	6/2015	3	Málaga	MML	No aplica	M0615ML2	No aplica	unspecified	unspecified	7	Aleta Amarilla	Thunnus albacares	Thunnus albacares
Mercas	6/2015	3	Málaga	MML	No aplica	M0615ML4	No aplica	unspecified	unspecified	7	Aleta Amarilla	Thunnus albacares	Thunnus albacares
Mercas	7/2015	3	Málaga	MML	No aplica	M0715ML4	No aplica	unspecified	unspecified	7	Aleta Amarilla	Thunnus albacares	Thunnus albacares
Mercas	8/2015	4	Málaga	MML	No aplica	M0815ML4	No aplica	unspecified	unspecified	7	Aleta Amarilla	Thunnus albacares	Thunnus albacares
Mercas	11/2014	1	Málaga	MML	No aplica	M1114ML3	No aplica	Thunnus obesus	Atún	7		Thunnus obesus	Thunnus obesus
Mercas	10/2015	4	Málaga	MML	No aplica	M1015ML1	No aplica	unspecified	unspecified	7,5	Patudo	Thunnus albacares	Thunnus Obesus
Mercas	5/2015	3	Málaga	MML	No aplica	M0515ML3	No aplica	unspecified	unspecified	7,5	Aleta Amarilla	Thunnus obesus	Thunnus albacares
Mercas	9/2015	4	Málaga	MML	No aplica	M0915ML4	No aplica	unspecified	unspecified	7,5	No sabe	Thunnus albacares	- sin especificar -
Mercas	5/2015	3	Málaga	MML	No aplica	M0515ML1	No aplica	Thunnus alabacares	Atún de aleta amarilla	7,5	Thunnus alabacares	Thunnus albacares	Thunnus albacares
Mercas	12/2014	1	Málaga	MML	No aplica	M1214ML2	No aplica	unspecified	Atún	7,5	Aleta Amarilla	Thunnus albacares	Thunnus albacares
Mercas	2/2015	2	Málaga	MML	No aplica	M0215ML1	No aplica	unspecified	Atún	7,5	Aleta Amarilla	Thunnus albacares	Thunnus albacares
Mercas	2/2015	2	Málaga	MML	No aplica	M0215ML3	No aplica	unspecified	Atún	7,5	Aleta Amarilla	Thunnus albacares	Thunnus albacares
Mercas	3/2015	2	Málaga	MML	No aplica	M0315ML2	No aplica	unspecified	unspecified	7,5	Aleta Amarilla	Thunnus albacares	Thunnus albacares
Mercas	9/2015	4	Málaga	MML	No aplica	M0915ML2	No aplica	unspecified	unspecified	7,5	Aleta Amarilla	Thunnus albacares	Thunnus albacares
Mercas	12/2014	1	Málaga	MML	No aplica	M1114ML4	No aplica	Thunnus alabacares	Atún	7,5		Thunnus albacares	Thunnus albacares
Gran superficie	12/2014	1	Barcelona	B0	No aplica	G1214BME	No aplica	Thunnus alabacares	Atún	7,5		Thunnus albacares	Thunnus albacares
Mercas	12/2014	1	Málaga	MML	No aplica	M1214ML1	No aplica	Thunnus obesus	Atún de ojo grande	7,5		Thunnus obesus	Thunnus obesus
Mercas	2/2015	2	Málaga	MML	No aplica	M0215ML4	No aplica	Thunnus alabacares	Atún de aleta amarilla	7,5		Thunnus albacares	Thunnus albacares
Mercas	4/2015	2	Málaga	MML	No aplica	M0415ML4	No aplica	Thunnus alabacares	Atún de aleta amarilla	7,5		Thunnus albacares	Thunnus albacares
Mercas	5/2015	3	Málaga	MML	No aplica	M0515ML4	No aplica	Thunnus obesus	Patudo	7,8	Thunnus obesus	Thunnus albacares	Thunnus obesus
Mercas	10/2015	4	Málaga	MML	No aplica	M1015ML3	No aplica	unspecified	unspecified	7,8	Aleta Amarilla	Thunnus obesus	Thunnus albacares
Mercas	10/2015	4	Málaga	MML	No aplica	M1015ML4	No aplica	unspecified	unspecified	7,8	Aleta Amarilla	Thunnus obesus	Thunnus albacares
Mercas	8/2015	4	Málaga	MML	No aplica	M0815ML3	No aplica	Thunnus alabacares	Atún de aleta amarilla	7,8		Thunnus obesus	Thunnus albacares
Mercas	7/2015	3	Málaga	MML	No aplica	M0715ML3	No aplica	Thunnus alabacares	Atún de aleta amarilla	7,8	Thunnus alabacares	Thunnus albacares	Thunnus albacares
Mercas	3/2015	2	Málaga	MML	No aplica	M0315ML4	No aplica	Thunnus alabacares	Atún	7,8		Thunnus albacares	Thunnus albacares
Mercas	1/2015	1	Málaga	MML	No aplica	M0115ML2	No aplica	Thunnus alabacares	Atún	8		Thunnus obesus	Thunnus albacares
Mercas	4/2015	2	Málaga	MML	No aplica	M0415ML1	No aplica	unspecified	Atún	8	Aleta Amarilla	Thunnus albacares	Thunnus albacares
Mercas	11/2014	1	Málaga	MML	No aplica	M1114ML1	No aplica	Thunnus alabacares	Atún	8		Thunnus albacares	Thunnus albacares
Mercas	11/2014	1	Málaga	MML	No aplica	M1114ML2	No aplica	Thunnus alabacares	Atún	8		Thunnus albacares	Thunnus albacares
Mercas	2/2015	2	Málaga	MML	No aplica	M0215ML2	No aplica	Thunnus alabacares	Rabil	8		Thunnus albacares	Thunnus albacares

Mercas	3/2015	2	Málaga	MML	No aplica	M0315ML3	No aplica	Thunnus alabacares	Atún de aleta amarilla	8		Thunnus albacares	Thunnus albacares
Mercas	9/2015	4	Málaga	MML	No aplica	M0915ML1	No aplica	unspecified	Atún	8,25	Aleta Amarilla	Thunnus albacares	Thunnus albacares
Mercas	12/2014	1	Málaga	MML	No aplica	M1214ML3	No aplica	Thunnus alabacares	Atún de aleta azul	8,3		Thunnus thynnus	Thunnus albacares
Mercas	10/2015	4	Málaga	MML	No aplica	M1015ML2	No aplica	unspecified	unspecified	8,3	Aleta Amarilla	Thunnus albacares	Thunnus albacares
Mercas	4/2015	2	Málaga	MML	No aplica	M0415ML3	No aplica	Thunnus alabacares	Atún de aleta amarilla	8,3		Thunnus albacares	Thunnus albacares
Mercas	1/2015	1	Málaga	MML	No aplica	M0115ML4	No aplica	Thunnus alabacares	Atún de aleta amarilla	8,5		Thunnus obesus	Thunnus albacares
Mercas	1/2015	1	Málaga	MML	No aplica	M0115ML1	No aplica	unspecified	Atún	8,5	Aleta Amarilla	Thunnus albacares	Thunnus albacares
Mercas	1/2015	1	Málaga	MML	No aplica	M0115ML3	No aplica	Thunnus obesus	Atún de ojo grande	8,5		Thunnus obesus	Thunnus obesus
Gran superficie	9/2015	4	Girona	G0	No aplica	G0915GCI	No aplica	unspecified	Tonyina	8,95	Thunnus Obesus	Thunnus obesus	Thunnus Obesus
Pescadería	6/2015	3	Barcelona	B2	No aplica	P0615B20	No aplica	unspecified	unspecified	9	No sabe	Thunnus thynnus	- sin especificar -
Gran superficie	12/2014	1	Barcelona	B0	No aplica	G1214BCR	No aplica	Thunnus alabacares	Atún	9		Thunnus albacares	Thunnus albacares
Mercas	4/2015	2	Málaga	MML	No aplica	M0415ML2	No aplica	Thunnus alabacares	Atún de aleta amarilla	9		Thunnus albacares	Thunnus albacares
Mercas	6/2015	3	Málaga	MML	No aplica	M0615ML1	No aplica	unspecified	Atún de ojo grande	10	Patudo	Thunnus thynnus	Thunnus obesus
Gran superficie	9/2015	4	Tarragona	T0	No aplica	G0915TCR	No aplica	Thunnus alabacares	Atún	10,9		Thunnus obesus	Thunnus albacares
Gran superficie	9/2015	4	Tarragona	T0	No aplica	G0915TCI	No aplica	Thunnus alabacares	Tonyina	10,9		Thunnus albacares	Thunnus albacares
Pescadería	4/2015	2	Tarragona	T4	No	P0415T40	No	unspecified	Atún	11,9	Rojo	Thunnus albacares	Thunnus thynnus
Gran superficie	3/2015	2	Barcelona	B0	No aplica	G0315BCI	No aplica	Thunnus alabacares	Atún	11,9		Thunnus albacares	Thunnus albacares
Mercas	11/2014	1	Barcelona	MBC	No aplica	M1114BC4	No aplica	unspecified	unspecified	12	"desconocido"	Thunnus albacares	- sin especificar -
Gran superficie	3/2015	2	Tarragona	T0	No aplica	G0315TCR	No aplica	unspecified	Atún	12	Yellow Fin	Thunnus albacares	Thunnus albacares
Mercas	8/2015	4	Barcelona	MBC	No	M0815BC2	Sí	unspecified	unspecified	12,5	Atún rojo	Thunnus obesus	Thunnus thynnus
Gran superficie	11/2014	1	Barcelona	B0	No	G1114BCI	No	unspecified	Atún	12,9	Atún Rojo	Thunnus albacares	Thunnus thynnus
Gran superficie	3/2015	2	Tarragona	T0	No aplica	G0315TCI	No aplica	Thunnus alabacares	Atún	12,9		Thunnus albacares	Thunnus albacares
Gran superficie	12/2014	1	Barcelona	B0	No aplica	G1214BCP	No aplica	unspecified	Tonyina	12,95	Aleta amarilla	Thunnus albacares	Thunnus albacares
Mercas	7/2015	3	Barcelona	MBC	No	M0715BC1	Sí	unspecified	unspecified	13	Atún Rojo	Thunnus obesus	Thunnus thynnus
Gran superficie	3/2015	2	Girona	G0	No aplica	G0315GCR	No aplica	Thunnus alabacares	Atún de aleta amarilla	13,2		Thunnus albacares	Thunnus albacares
Gran superficie	3/2015	2	Barcelona	B0	No aplica	G0315BCR	No aplica	Thunnus alabacares	Atún	13,99		Thunnus albacares	Thunnus albacares
Mercas	2/2015	2	Barcelona	MBC	No	M0215BC3	Sí	unspecified	Atún	14	Rojo	Thunnus albacares	Thunnus thynnus
Mercas	7/2015	3	Barcelona	MBC	No	M0715BC2	Sí	unspecified	unspecified	14,5	Atún Rojo	Thunnus obesus	Thunnus thynnus
Gran superficie	12/2014	1	Barcelona	B0	No aplica	G1214BCI	No aplica	unspecified	Atún	14,5	Aleta amarilla	Thunnus albacares	Thunnus thynnus
Gran superficie	12/2014	1	Tarragona	T0	No aplica	G1214TCI	No aplica	Thunnus alabacares	Atún	14,5		Thunnus albacares	Thunnus albacares
Mercas	4/2015	2	Madrid	MMD	No	M0415MD:	Sí	unspecified	unspecified	15	Rojo	Thunnus obesus	Thunnus thynnus
Mercas	9/2015	4	Barcelona	MBC	No	M0915BC1	Sí	unspecified	unspecified	15	Rojo	Thunnus obesus	Thunnus thynnus
Mercas	11/2014	1	Barcelona	MBC	No aplica	M1114BC3	No aplica	unspecified	unspecified	15	BLUEFIN	Thunnus albacares	Thunnus thynnus
Mercas	6/2015	3	Barcelona	MBC	No	M0615BC4	Sí	unspecified	unspecified	15	Atún Rojo	Thunnus obesus	Thunnus obesus
Mercas	7/2015	3	Barcelona	MBC	No	M0715BC3	Sí	unspecified	unspecified	15	Atún Rojo	Thunnus obesus	Thunnus thynnus
Mercas	8/2015	4	Barcelona	MBC	No	M0815BC1	Sí	unspecified	unspecified	15	Atún Rojo	Thunnus obesus	Thunnus thynnus
Mercas	8/2015	4	Barcelona	MBC	No	M0815BC4	Sí	unspecified	unspecified	15	Atún Rojo	Thunnus obesus	Thunnus thynnus
Pescadería	9/2015	4	Girona	G4	No aplica	P0915G40	No aplica	unspecified	unspecified	15	Aleta Amarilla	Thunnus obesus	Thunnus albacares
Mercas	1/2015	1	Barcelona	MBC	No aplica	M0115BC4	No aplica	unspecified	Patudo	15		Thunnus albacares	Thunnus obesus
Gran superficie	9/2015	4	Girona	G0	No aplica	G0915GCR	No aplica	Thunnus alabacares	Tonyina	15		Thunnus obesus	Thunnus albacares
Mercas	5/2015	3	Barcelona	MBC	No	M0515BC2	Sí	unspecified	unspecified	15	- sin especificar -	Thunnus thynnus	- sin especificar -

Mercas	3/2015	2	Barcelona	MBC	No	M0315BC2	Sí	unspecified	unspecified	15	Rojo	Thunnus thynnus	Thunnus thynnus
Mercas	3/2015	2	Málaga	MML	No	M0315ML1	Sí	unspecified	unspecified	15	Rojo	Thunnus thynnus	Thunnus thynnus
Mercas	11/2014	1	Barcelona	MBC	No aplica	M1114BC2	No aplica	unspecified	unspecified	15	Blue Fin	Thunnus thynnus	Thunnus thynnus
Pescadería	4/2015	2	Girona	G3	No aplica	P0415G32	No aplica	unspecified	Atún	15	Albacares	Thunnus albacares	Thunnus albacares
Pescadería	2/2015	2	Tarragona	T3	No aplica	P0215T30	No aplica	unspecified	Tonyina	15,9	Aleta Amarilla	Thunnus albacares	Thunnus albacares
Pescadería	4/2015	2	Tarragona	T2	No aplica	P0415T20	No aplica	Thunnus alabacares	Tonyina	15,9		Thunnus albacares	Thunnus albacares
Mercas	5/2015	3	Barcelona	MBC	No	M0515BC4	Sí	unspecified	unspecified	16	Atún Rojo	Thunnus obesus	Thunnus thynnus
Mercas	5/2015	3	Barcelona	MBC	No	M0515BC1	No	unspecified	unspecified	16	Atún Rojo	Thunnus thynnus	Thunnus thynnus
Pescadería	6/2015	3	Tarragona	T2	No aplica	P0615T20	No aplica	unspecified	unspecified	16,8	Yellow fin	Thunnus thynnus	Thunnus albacares
Pescadería	1/2015	1	Girona	G4	No aplica	P0115G40	No aplica	unspecified	Tonyina	16,95	Bonito	Thunnus obesus	Thunnus alalunga
Pescadería	12/2014	1	Girona	G4	No aplica	P1214G40	No aplica	unspecified	Tonyina	16,95	Desconocido	Thunnus obesus	- sin especificar -
Pescadería	8/2015	4	Tarragona	T2	No aplica	P0815T20	No aplica	Thunnus alabacares	Tonyina	16,95		Thunnus albacares	Thunnus albacares
Mercas	6/2015	3	Barcelona	MBC	No	M0615BC2	Sí	unspecified	unspecified	17	Atún rojo	Thunnus obesus	Thunnus thynnus
Mercas	12/2014	1	Barcelona	MBC	No	M1214BC2	Sí	unspecified	Atún	17	atun rojo	Thunnus albacares	Thunnus thynnus
Mercas	12/2014	1	Barcelona	MBC	No	M1214BC1	Sí	unspecified	Atún	17	Atun rojo	Thunnus albacares	Thunnus thynnus
Pescadería	2/2015	2	Girona	G2	No aplica	P0215G20	No aplica	unspecified	Tonyina	17	No sabe	Thunnus albacares	- sin especificar -
Mercas	8/2015	4	Málaga	MML	No aplica	M0815ML2	No aplica	unspecified	unspecified	17	Aleta Amarilla	Thunnus albacares	Thunnus albacares
Mercas	11/2014	1	Barcelona	MBC	No aplica	M1114BC1	No aplica	unspecified	unspecified	17	"Atún rojo del buen	Thunnus thynnus	Thunnus thynnus
Mercas	9/2015	4	Barcelona	MBC	No	M0915BC3	Sí	unspecified	unspecified	17,5	Rojo	Thunnus obesus	Thunnus thynnus
Pescadería	5/2015	3	Tarragona	T2	No aplica	P0515T20	No aplica	Thunnus alabacares	Tonyina	17,95	Thunnus alabacares	Thunnus albacares	Thunnus albacares
Mercas	4/2015	2	Barcelona	MBC	No	M0415BC4	Sí	unspecified	Atún	18	Rojo	Thunnus albacares	Thunnus thynnus
Mercas	6/2015	3	Málaga	MML	No aplica	M0615ML3	No aplica	unspecified	Atún de ojo grande	18	patudo	Thunnus thynnus	Thunnus obesus
Mercas	8/2015	4	Barcelona	MBC	No	M0815BC3	Sí	unspecified	unspecified	18	Atún Rojo	Thunnus albacares	Thunnus thynnus
Mercas	6/2015	3	Barcelona	MBC	No	M0615BC1	Sí	unspecified	unspecified	18	Atun rojo	Thunnus obesus	Thunnus thynnus
Pescadería	1/2015	1	Barcelona	B3	No aplica	P0115B30	No aplica	unspecified	Atún	18	Aleta Amarilla	Thunnus obesus	Thunnus albacares
Mercas	10/2015	4	Barcelona	MBC	No	M1015BC2	Sí	unspecified	unspecified	18		Thunnus albacares	- sin especificar -
Pescadería	6/2015	3	Tarragona	T1	No aplica	P0615T10	No aplica	unspecified	unspecified	18	Yellow fin	Thunnus albacares	Thunnus albacares
Mercas	1/2015	1	Barcelona	MBC	No	M0115BC1	Sí	unspecified	Atún	18	Rojo	Thunnus thynnus	Thunnus thynnus
Mercas	1/2015	1	Barcelona	MBC	No	M0115BC2	Sí	unspecified	Atún	18	Rojo	Thunnus thynnus	Thunnus thynnus
Mercas	3/2015	2	Barcelona	MBC	No	M0315BC3	Sí	unspecified	unspecified	18	Rojo	Thunnus thynnus	Thunnus thynnus
Mercas	5/2015	3	Barcelona	MBC	No	M0515BC3	Sí	unspecified	unspecified	18	Atún Rojo	Thunnus thynnus	Thunnus thynnus
Mercas	12/2014	1	Barcelona	MBC	No	M1214BC4	No	unspecified	Atún	18	Atun rojo	Thunnus thynnus	Thunnus thynnus
Mercas	12/2014	1	Barcelona	MBC	No	M1214BC3	Sí	unspecified	Atún	18,5	Blue fin	Thunnus albacares	Thunnus thynnus
Pescadería	1/2015	1	Tarragona	T3	No	P0115T30	No	unspecified	unspecified	18,5	Atún rojo salvaje (nc	Thunnus albacares	Thunnus thynnus
Pescadería	5/2015	3	Girona	G3	No	P0515G30	No	unspecified	Atún	18,9	Atún rojo	Thunnus albacares	Thunnus thynnus
Pescadería	10/2015	4	Girona	G4	No aplica	P1015G40	No aplica	unspecified	Atún	18,9	Yellow Fin	Thunnus albacares	Thunnus albacares
Pescadería	1/2015	1	Tarragona	T2	No aplica	P0115T20	No aplica	Thunnus alabacares	Tonyina	18,9		Thunnus albacares	Thunnus albacares
Pescadería	3/2015	2	Girona	G3	No	P0315G30	No	unspecified	unspecified	19	atún rojo	Thunnus obesus	Thunnus thynnus
Mercas	10/2015	4	Barcelona	MBC	No	M1015BC1	Sí	unspecified	unspecified	19	Rojo	Thunnus thynnus	Thunnus thynnus
Pescadería	3/2015	2	Tarragona	T1	No	P0315T10	No	unspecified	Atún	19,75	Rojo	Thunnus obesus	Thunnus thynnus
Pescadería	1/2015	1	Barcelona	B2	No	P0115B20	No	unspecified	unspecified	19,8	Rojo	Thunnus albacares	Thunnus thynnus

Pescadería	6/2015	3	Girona	G3	No	P0615G30	No	unspecified	Atún	19,8	ATUN ROJO	Thunnus obesus	Thunnus thynnus
Gran superficie	6/2015	3	Tarragona	T0	No	G0615TCI	No	unspecified	Atún rojo	19,95	Atún rojo	Thunnus thynnus	Thunnus thynnus
Pescadería	10/2015	4	Barcelona	B3	No aplica	P1015B30	No aplica	unspecified	unspecified	19,99	Yellow Fin	Thunnus albacares	Thunnus albacares
Mercas	10/2015	4	Barcelona	MBC	No	M1015BC3	Sí	unspecified	unspecified	20	Rojo	Thunnus obesus	Thunnus thynnus
Mercas	1/2015	1	Barcelona	MBC	No aplica	M0115BC3	No aplica	unspecified	Patudo	20		Thunnus thynnus	Thunnus obesus
Pescadería	12/2014	1	Girona	G3	No	P1214G30	No	Thunnus thynnus	Atún rojo	20		Thunnus albacares	Thunnus thynnus
Mercas	12/2014	1	Madrid	MMD	No	M1114MD:	Sí	Thunnus thynnus	unspecified	20		Thunnus albacares	Thunnus thynnus
Mercas	4/2015	2	Barcelona	MBC	No	M0415BC3	Sí	unspecified	Atún	20	Rojo	Thunnus thynnus	Thunnus thynnus
Pescadería	8/2015	4	Barcelona	B4	No aplica	P0815B40	No aplica	unspecified	Tonyina	20,99	No sabe	Thunnus obesus	- sin especificar -
Pescadería	1/2015	1	Girona	G2	No aplica	P0115G20	No aplica	unspecified	Tonyina	21	Aleta Amarilla (colo	Thunnus thynnus	Thunnus albacares
Mercas	3/2015	2	Barcelona	MBC	No	M0315BC1	Sí	unspecified	Atún	21	Rojo	Thunnus thynnus	Thunnus thynnus
Mercas	3/2015	2	Barcelona	MBC	No	M0315BC4	Sí	unspecified	unspecified	21	Rojo	Thunnus thynnus	Thunnus thynnus
Pescadería	7/2015	3	Barcelona	B4	No	P0715B40	No	unspecified	unspecified	21,4	Rojo	Thunnus thynnus	Thunnus thynnus
Pescadería	5/2015	3	Barcelona	B4	No	P0515B40	No	unspecified	unspecified	21,8	Rojo	Thunnus thynnus	Thunnus thynnus
Pescadería	7/2015	3	Girona	G3	No	P0715G30	No	unspecified	unspecified	21,8	ATUN ROJO	Thunnus thynnus	Thunnus thynnus
Mercas	1/2015	1	Madrid	MMD	No	M0115MD:	No	unspecified	Atún	22	Rojo	Thunnus albacares	Thunnus thynnus
Mercas	7/2015	3	Barcelona	MBC	No	M0715BC4	Sí	unspecified	unspecified	22	Atún Rojo	Thunnus obesus	Thunnus thynnus
Mercas	4/2015	2	Madrid	MMD	No aplica	M0415MD:	No aplica	unspecified	unspecified	22	ATUN GORDO PATU	Thunnus albacares	Thunnus obesus
Mercas	9/2015	4	Málaga	MML	No aplica	M0915ML3	No aplica	unspecified	Atún de ojo grande	22		Thunnus thynnus	Thunnus Obesus
Pescadería	6/2015	3	Barcelona	B3	No aplica	P0615B30	No aplica	unspecified	unspecified	22	no saben	Thunnus thynnus	- sin especificar -
Pescadería	5/2015	3	Girona	G4	No aplica	P0515G40	No aplica	unspecified	unspecified	22	No sabe	Thunnus albacares	- sin especificar -
Mercas	4/2015	2	Barcelona	MBC	No	M0415BC2	Sí	unspecified	Atún	22		Thunnus albacares	- sin especificar -
Mercas	1/2015	1	Madrid	MMD	No	M0115MD:	No	unspecified	Atún	22	Rojo	Thunnus thynnus	Thunnus thynnus
Pescadería	3/2015	2	Barcelona	B3	No	P0315B30	No	unspecified	unspecified	22	Rojo	Thunnus thynnus	Thunnus thynnus
Mercas	9/2015	4	Barcelona	MBC	No	M0915BC2	Sí	unspecified	unspecified	22	Rojo	Thunnus thynnus	Thunnus thynnus
Mercas	9/2015	4	Barcelona	MBC	No	M0915BC4	Sí	unspecified	unspecified	22	Rojo	Thunnus thynnus	Thunnus thynnus
Pescadería	4/2015	2	Girona	G3	No aplica	P0415G31	No aplica	unspecified	Atún	22	Albacares	Thunnus albacares	Thunnus albacares
Mercas	1/2015	1	Madrid	MMD	No	M0115MD:	No	unspecified	Atún rojo	22		Thunnus thynnus	Thunnus thynnus
Mercas	1/2015	1	Madrid	MMD	No	M0115MD4	No	unspecified	Atún rojo	22		Thunnus thynnus	Thunnus thynnus
Mercas	10/2015	4	Madrid	MMD	No	M1015MD:	Sí	Thunnus thynnus	Atún	22		Thunnus thynnus	Thunnus thynnus
Mercas	12/2014	1	Madrid	MMD	No	M1214MD:	Sí	Thunnus thynnus	Atún rojo	22,5		Thunnus albacares	Thunnus thynnus
Pescadería	4/2015	2	Girona	G4	No	P0415G40	No	unspecified	Tonyina	22,9	Rojo	Thunnus thynnus	Thunnus thynnus
Pescadería	2/2015	2	Tarragona	T1	No	P0215T10	Sí	unspecified	unspecified	22,95	Vermella del Medite	Thunnus albacares	Thunnus thynnus
Pescadería	10/2015	4	Girona	G2	No	P1015G20	No	unspecified	unspecified	23	Rojo	Thunnus obesus	Thunnus thynnus
Mercas	12/2014	1	Madrid	MMD	No	M1214MD:	Sí	Thunnus thynnus	unspecified	23		Thunnus albacares	- sin especificar -
Mercas	2/2015	2	Barcelona	MBC	No	M0215BC4	Sí	unspecified	Atún	23	Rojo	Thunnus thynnus	Thunnus thynnus
Mercas	2/2015	2	Madrid	MMD	No	M0215MD:	Sí	unspecified	Atún	23	Rojo	Thunnus thynnus	Thunnus thynnus
Mercas	4/2015	2	Madrid	MMD	No	M0315MD:	Sí	unspecified	unspecified	23	Rojo	Thunnus thynnus	Thunnus thynnus
Mercas	4/2015	2	Barcelona	MBC	No	M0415BC1	Sí	unspecified	Atún	23	Rojo	Thunnus thynnus	Thunnus thynnus
Mercas	12/2014	1	Madrid	MMD	No	M1114MD:	Sí	unspecified	Atún rojo	23		Thunnus thynnus	Thunnus thynnus
Mercas	12/2014	1	Madrid	MMD	No	M1214MD:	Sí	unspecified	Atún rojo	23,5		Thunnus thynnus	Thunnus thynnus

Pescadería	2/2015	2	Girona	G4	No	P0215G40	No	unspecified	unspecified	24	Rojo	Thunnus albacares	Thunnus thynnus
Pescadería	6/2015	3	Girona	G4	No	P0615G40	No	unspecified	unspecified	24	rojo	Thunnus obesus	Thunnus thynnus
Pescadería	10/2015	4	Barcelona	B4	No	P1015B40	No	unspecified	Atún	24	Rojo	Thunnus albacares	Thunnus thynnus
Pescadería	10/2015	4	Tarragona	T4	No	P1015T40	No	unspecified	Atún	24	Rojo	Thunnus albacares	Thunnus thynnus
Pescadería	8/2015	4	Girona	G2	No	P0815G20	Sí	unspecified	Tonyina	24	Rojo	Thunnus obesus	Thunnus thynnus
Mercas	9/2015	4	Madrid	MMD	No aplica	M0915MD	No aplica	unspecified	unspecified	24	Patudo	Thunnus thynnus	Thunnus Obesus
Mercas	4/2015	2	Madrid	MMD	No aplica	M0415MD	No aplica	unspecified	unspecified	24	BIGEYE	Thunnus thynnus	Thunnus obesus
Pescadería	4/2015	2	Barcelona	B4	No	P0415B40	Sí	unspecified	Tonyina	24	atún rojo	Thunnus thynnus	Thunnus thynnus
Pescadería	11/2014	1	Barcelona	B4	No	P1114B40	No	unspecified	Atún	24	"De playa"	Thunnus thynnus	Thunnus thynnus
Mercas	3/2015	2	Madrid	MMD	No	M0315MD	Sí	unspecified	Atún rojo	24		Thunnus thynnus	Thunnus thynnus
Mercas	3/2015	2	Madrid	MMD	No	M0315MD	Sí	unspecified	Atún rojo	24		Thunnus thynnus	Thunnus thynnus
Mercas	10/2015	4	Madrid	MMD	No	M1015MD	Sí	Thunnus thynnus	Atún rojo	24		Thunnus thynnus	Thunnus thynnus
Pescadería	2/2015	2	Tarragona	T2	No	P0215T20	Sí	unspecified	unspecified	24,5	Rojo	Thunnus albacares	Thunnus thynnus
Mercas	12/2014	1	Madrid	MMD	No	M1214MD	Sí	unspecified	Atún rojo	24,5		Thunnus albacares	Thunnus thynnus
Mercas	12/2014	1	Madrid	MMD	No	M1114MD	Sí	unspecified	Atún rojo	24,75		Thunnus albacares	Thunnus thynnus
Pescadería	7/2015	3	Tarragona	T4	No	P0715T40	No	unspecified	unspecified	24,8	Rojo	Thunnus obesus	Thunnus thynnus
Pescadería	11/2014	1	Tarragona	T1	No	P1114T10	Sí	unspecified	unspecified	24,8	Atún Rojo	Thunnus albacares	Thunnus thynnus
Pescadería	1/2015	1	Tarragona	T4	No	P0115T40	No	unspecified	unspecified	24,8	No sabe, presumible	Thunnus albacares	- sin especificar -
Pescadería	3/2015	2	Barcelona	B1	No	P0315B10	Sí	unspecified	unspecified	25	Rojo	Thunnus albacares	Thunnus thynnus
Mercas	10/2015	4	Barcelona	MBC	No	M1015BC4	Sí	unspecified	unspecified	25	Rojo	Thunnus albacares	Thunnus thynnus
Pescadería	12/2014	1	Barcelona	B2	No	P1214B20	Sí	unspecified	unspecified	25	Blue Fin del Atlántic	Thunnus albacares	Thunnus thynnus
Mercas	2/2015	2	Madrid	MMD	No	M0215MD	Sí	unspecified	Atún rojo	25		Thunnus albacares	Thunnus thynnus
Mercas	2/2015	2	Barcelona	MBC	No	M0215BC2	Sí	unspecified	Atún	25		Thunnus thynnus	- sin especificar -
Mercas	2/2015	2	Barcelona	MBC	No	M0215BC1	Sí	unspecified	Atún	25	Rojo	Thunnus thynnus	Thunnus thynnus
Mercas	2/2015	2	Madrid	MMD	No	M0215MD	Sí	unspecified	Atún	25	Rojo	Thunnus thynnus	Thunnus thynnus
Mercas	9/2015	4	Madrid	MMD	No	M0915MD	Sí	unspecified	unspecified	25	Rojo	Thunnus thynnus	Thunnus thynnus
Mercas	6/2015	3	Barcelona	MBC	No	M0615BC3	Sí	unspecified	unspecified	25	Atún rojo	Thunnus thynnus	Thunnus thynnus
Mercas	3/2015	2	Madrid	MMD	No	M0315MD	Sí	unspecified	Atún rojo	25		Thunnus thynnus	Thunnus thynnus
Pescadería	8/2015	4	Girona	G1	No	P0815G10	Sí	unspecified	unspecified	26	Rojo	Thunnus albacares	Thunnus thynnus
Pescadería	12/2014	1	Girona	G1	No	P1214G10	Sí	unspecified	unspecified	26	"del atlántico", supu	Thunnus albacares	Thunnus thynnus
Pescadería	11/2014	1	Barcelona	B1	No aplica	P1114B10	No aplica	unspecified	Atún	26	Desconocido	Thunnus albacares	- sin especificar -
Pescadería	6/2015	3	Girona	G1	No aplica	P0615G10	No aplica	unspecified	unspecified	26	- sin especificar -	Thunnus albacares	- sin especificar -
Mercas	10/2015	4	Madrid	MMD	No	M1015MD	Sí	unspecified	unspecified	26	Rojo	Thunnus thynnus	Thunnus thynnus
Mercas	12/2014	1	Madrid	MMD	No	M1114MD	Sí	unspecified	Atún rojo	26		Thunnus thynnus	Thunnus thynnus
Pescadería	5/2015	3	Tarragona	T3	No	P0515T30	Sí	unspecified	unspecified	26,8	Rojo	Thunnus albacares	Thunnus thynnus
Pescadería	8/2015	4	Tarragona	T3	No	P0815T30	No	unspecified	unspecified	26,8	Rojo	Thunnus thynnus	Thunnus thynnus
Pescadería	7/2015	3	Tarragona	T3	No	P0715T30	Sí	unspecified	unspecified	26,9	Rojo	Thunnus albacares	Thunnus thynnus
Pescadería	3/2015	2	Barcelona	B4	No	P0315B40	Sí	unspecified	Tonyina	26,9	Rojo	Thunnus thynnus	Thunnus thynnus
Mercas	8/2015	4	Madrid	MMD	No	M0815MD	Sí	unspecified	unspecified	27	Rojo	Thunnus thynnus	Thunnus thynnus
Pescadería	10/2015	4	Tarragona	T1	No	P1015T10	No	unspecified	Atún	28	Rojo	Thunnus albacares	Thunnus thynnus
Pescadería	3/2015	2	Girona	G4	No aplica	P0315G40	No aplica	unspecified	unspecified	28	No sabe	Thunnus albacares	- sin especificar -

Mercas	8/2015	4	Madrid	MMD	No	M0815MD	Sí	unspecified	unspecified	28	Rojo	Thunnus thynnus	Thunnus thynnus
Mercas	9/2015	4	Madrid	MMD	No	M0915MD	Sí	unspecified	unspecified	28	Rojo	Thunnus thynnus	Thunnus thynnus
Pescadería	8/2015	4	Girona	G4	No	P0815G40	Sí	unspecified	unspecified	28	Rojo	Thunnus thynnus	Thunnus thynnus
Pescadería	11/2014	1	Girona	G4	No aplica	P1114G40	No aplica	unspecified	unspecified	28	"de aqui"	Thunnus thynnus	Thunnus thynnus
Pescadería	4/2015	2	Girona	G3	No	P0415G30	No	unspecified	Atún de aleta azul	28		Thunnus thynnus	Thunnus thynnus
Pescadería	12/2014	1	Barcelona	B3	No	P1214B30	No	unspecified	unspecified	28,5	Atun rojo	Thunnus albacares	Thunnus thynnus
Pescadería	8/2015	4	Tarragona	T4	No	P0815T40	No	unspecified	Atún	29	Rojo	Thunnus albacares	Thunnus thynnus
Pescadería	2/2015	2	Barcelona	B4	No	P0215B40	No	unspecified	Atún	29,5	Atun rojo	Thunnus thynnus	Thunnus thynnus
Pescadería	11/2014	1	Tarragona	T4	No aplica	P1114T40	No aplica	unspecified	unspecified	29,6	"Atún de aquí"	Thunnus thynnus	Thunnus thynnus
Pescadería	3/2015	2	Tarragona	T4	No	P0315T40	Sí	unspecified	unspecified	29,9	Rojo	Thunnus albacares	Thunnus thynnus
Pescadería	2/2015	2	Girona	G3	No	P0215G30	Sí	unspecified	Atún	29,9	atún rojo	Thunnus albacares	Thunnus thynnus
Pescadería	2/2015	2	Tarragona	T4	No aplica	P0215T40	No aplica	unspecified	Tonyina	29,9	Yellow Fin	Thunnus albacares	Thunnus albacares
Pescadería	8/2015	4	Girona	G3	No	P0815G30	No	unspecified	unspecified	30	Atún rojo	Thunnus obesus	Thunnus thynnus
Mercas	7/2015	3	Madrid	MMD	No	M0715MD	Sí	unspecified	unspecified	30	- sin especificar -	Thunnus thynnus	- sin especificar -
Pescadería	1/2015	1	Barcelona	B4	No	P0115B40	Sí	unspecified	unspecified	30	Rojo, de la costa	Thunnus thynnus	Thunnus thynnus
Mercas	2/2015	2	Madrid	MMD	No	M0215MD	Sí	unspecified	Atún	30	Rojo	Thunnus thynnus	Thunnus thynnus
Mercas	7/2015	3	Madrid	MMD	No	M0715MD	Sí	unspecified	unspecified	30	Rojo	Thunnus thynnus	Thunnus thynnus
Mercas	8/2015	4	Madrid	MMD	No	M0815MD	Sí	unspecified	unspecified	31	Rojo	Thunnus thynnus	Thunnus thynnus
Mercas	10/2015	4	Madrid	MMD	No	M1015MD	Sí	unspecified	Atún rojo	31		Thunnus thynnus	Thunnus thynnus
Pescadería	9/2015	4	Tarragona	T4	No	P0915T40	No	unspecified	Atún	32	Rojo	Thunnus obesus	Thunnus thynnus
Pescadería	12/2014	1	Tarragona	T4	No	P1214T40	No	unspecified	Atún	32	Blue fin	Thunnus albacares	Thunnus thynnus
Pescadería	1/2015	1	Girona	G3	No	P0115G30	No	unspecified	Tonyina	32	Atún rojo	Thunnus albacares	Thunnus thynnus
Pescadería	12/2014	1	Barcelona	B4	No	P1214B40	No	unspecified	unspecified	32	Atún Rojo	Thunnus albacares	Thunnus thynnus
Mercas	4/2015	2	Madrid	MMD	No	M0415MD	Sí	unspecified	unspecified	32	Rojo	Thunnus thynnus	Thunnus thynnus
Pescadería	3/2015	2	Girona	G2	No	P0315G20	Sí	unspecified	Tonyina	32	Rojo	Thunnus thynnus	Thunnus thynnus
Pescadería	8/2015	4	Barcelona	B2	No	P0815B20	No	unspecified	unspecified	32	Atún Rojo	Thunnus thynnus	Thunnus thynnus
Pescadería	5/2015	3	Barcelona	B1	No	P0515B10	No	unspecified	Tonyina	33	Rojo	Thunnus thynnus	Thunnus thynnus
Mercas	8/2015	4	Madrid	MMD	No	M0815MD	Sí	unspecified	unspecified	33	Rojo	Thunnus thynnus	Thunnus thynnus
Mercas	9/2015	4	Madrid	MMD	No	M0915MD	Sí	unspecified	unspecified	33	Rojo	Thunnus thynnus	Thunnus thynnus
Pescadería	2/2015	2	Barcelona	B1	No	P0215B10	Sí	unspecified	Tonyina	33	Roja de la costa	Thunnus thynnus	Thunnus thynnus
Gran superficie	12/2014	1	Girona	G0	No aplica	G1214GCI	No aplica	Thunnus alabacares	Tonyina	33,9		Thunnus albacares	Thunnus albacares
Gran superficie	3/2015	2	Girona	G0	No aplica	G0315GCI	No aplica	Thunnus alabacares	Tonyina	34,9		Thunnus albacares	Thunnus albacares
Pescadería	5/2015	3	Barcelona	B2	No	P0515B20	Sí	unspecified	unspecified	35	Rojo	Thunnus obesus	Thunnus thynnus
Pescadería	2/2015	2	Barcelona	B3	No	P0215B30	No	unspecified	Atún	35,5	Rojo	Thunnus thynnus	Thunnus thynnus
Pescadería	5/2015	3	Tarragona	T4	No	P0515T40	No	unspecified	unspecified	36	Rojo	Thunnus thynnus	Thunnus thynnus
Pescadería	11/2014	1	Girona	G1	No aplica	P1114G10	No aplica	unspecified	unspecified	36	"Atún del bueno"	Thunnus thynnus	Thunnus thynnus
Gran superficie	6/2015	3	Girona	G0	No	G0615GCI	Sí	Thunnus thynnus	Atún rojo	38	Thunnus thynnus	Thunnus obesus	Thunnus thynnus
Pescadería	3/2015	2	Barcelona	B2	No	P0315B20	No	unspecified	unspecified	38	Rojo	Thunnus thynnus	Thunnus thynnus
Pescadería	10/2015	4	Barcelona	B2	No	P1015B20	No	unspecified	unspecified	39	Rojo	Thunnus thynnus	Thunnus thynnus
Pescadería	3/2015	2	Girona	G1	No	P0315G10	Sí	unspecified	unspecified	39,8	Rojo	Thunnus thynnus	Thunnus thynnus
Pescadería	5/2015	3	Girona	G1	No	P0515G10	Sí	unspecified	unspecified	40	Rojo (ventresca)	Thunnus thynnus	Thunnus thynnus
