# Supplementary material for: Tuna Species Substitution in the Spanish Commercial Chain: A Knock-On Effect
Source: PLoS One. 2017 Jan 26;12(1):e0170809. doi: 10.1371/journal.pone.0170809 (PMC5268641; doi:10.1371/journal.pone.0170809)
Supplement: S3 File — (PDF) [file pone.0170809.s004.pdf]

muestra	MesAño	Q	Provincia	Zona	DCA	Código	Conocen DCA	Identificac	plato	Resultados	Sp declarada
Rest	11/2014	1	Girona	G2	No	R1114G21	Sí	asked	cooked	Thunnus thynnus	Thunnus thynnus
Rest	3/2015	2	Girona	G1	No	R0315G11	No	asked	cooked	Thunnus thynnus	Thunnus thynnus
Rest	4/2015	2	Girona	G3	No	R0415G31	No	asked	cooked	Thunnus thynnus	Thunnus thynnus
Rest	5/2015	3	Tarragona	T2	No	R0515T21	No	asked	cooked	Thunnus thynnus	Thunnus thynnus
Rest	10/09/2015	4	Tarragona	T3	Sí	R0915T31	Sí	asked	cooked	Thunnus thynnus	Thunnus thynnus
Rest	09/09/2015	4	Tarragona	T4	No	R0915T41	No	asked	cooked	Thunnus thynnus	Thunnus thynnus
Rest	11/2014	1	Barcelona	B4	No	R1114B41	No	asked	cooked	Thunnus albacares	Thunnus albacares
Rest	12/2014	1	Girona	G3	No aplica	R1214G31	No aplica	asked	cooked	Thunnus albacares	Thunnus albacares
Rest	1/2015	1	Girona	G3	No aplica	R0115G31	No aplica	asked	cooked	Thunnus albacares	Thunnus albacares
Rest	2/2015	2	Barcelona	B4	No aplica	R0215B42	No aplica	asked	cooked	Thunnus albacares	Thunnus albacares
Rest	3/2015	2	Tarragona	T1	No aplica	R0315T11	No aplica	asked	cooked	Thunnus albacares	Thunnus albacares
Rest	3/2015	2	Girona	G3	No	R0315G31	No	asked	cooked	Thunnus obesus	Thunnus thynnus
Rest	6/2015	3	Girona	G1	No	R0615G11	No	asked	cooked	Thunnus obesus	Thunnus thynnus
Rest	14/10/2015	4	Barcelona	B4	No	R1015B41	No	asked	cooked	Thunnus obesus	Thunnus thynnus
Rest	31/10/2015	4	Girona	G4	No	R1015G41	No	asked	cooked	Thunnus obesus	Thunnus thynnus
Rest	11/2014	1	Barcelona	B1	No	R1114B11	Sí	asked	cooked	Thunnus albacares	Thunnus thynnus
Rest	11/2014	1	Girona	G1	No	R1114G11	No	asked	cooked	Thunnus albacares	Thunnus thynnus
Rest	11/2014	1	Girona	G3	No	R1114G31	No	asked	cooked	Thunnus albacares	Thunnus thynnus
Rest	5/2015	3	Tarragona	T4	No	R0515T41	Sí	asked	cooked	Thunnus albacares	Thunnus thynnus
Rest	6/2015	3	Barcelona	B3	No	R0615B31	No	asked	cooked	Thunnus albacares	Thunnus thynnus
Rest	21/08/2015	4	Girona	G4	No	R0815G41	No	asked	cooked	Thunnus albacares	Thunnus thynnus
Rest	05/09/2015	4	Girona	G2	No	R0915G21	No	asked	cooked	Thunnus albacares	Thunnus thynnus
Rest	15/08/2015	4	Tarragona	T2	No	R0815T21	Sí	asked	cooked	Thunnus albacares	Thunnus thynnus
Rest	14/08/2015	4	Tarragona	T4	No	R0815T41	No	asked	cooked	Thunnus albacares	Thunnus thynnus
Rest	21/09/2015	4	Girona	G3	No	R0915G31	No	asked	cooked	Thunnus albacares	Thunnus thynnus
Rest	1/2015	1	Barcelona	B4	No aplica	R0115B41	No aplica	asked	cooked	Thunnus obesus	Thunnus albacares
Rest	2/2015	2	Barcelona	B3	No aplica	R0215B31	No aplica	asked	cooked	Thunnus obesus	Thunnus albacares
Rest	2/2015	2	Tarragona	T1	No aplica	R0215T11	No aplica	asked	cooked	Thunnus obesus	Thunnus albacares
Rest	1/2015	1	Barcelona	B3	No	R0115B31	No	menu	cooked	Thunnus thynnus	Thunnus thynnus
Rest	7/2015	3	Girona	G3	No	R0715G31	No	menu	cooked	Thunnus thynnus	Thunnus thynnus
Rest	7/2015	3	Tarragona	T3	Sí	R0715T31	Sí	menu	cooked	Thunnus thynnus	Thunnus thynnus
Rest	15/08/2015	4	Tarragona	T3	No	R0815T31	Sí	menu	cooked	Thunnus thynnus	Thunnus thynnus
Rest	11/2014	1	Tarragona	T3	No	R1114T31	Sí	menu	cooked	Thunnus obesus	Thunnus thynnus

Rest	24/10/2015	4	Tarragona	T3	No	R1015T31	Sí	menu	cooked	Thunnus obesus	Thunnus thynnus
Rest	12/2014	1	Barcelona	B4	No	R1214B42	No	menu	cooked	Thunnus albacares	Thunnus thynnus
Rest	11/2014	1	Tarragona	T1	No	R1114T11	No	menu	cooked	Thunnus albacares	Thunnus thynnus
Rest	1/2015	1	Tarragona	T1	No	R0115T11	No	menu	cooked	Thunnus albacares	Thunnus thynnus
Rest	6/2015	3	Tarragona	T1	No	R0615T11	No	menu	cooked	Thunnus albacares	Thunnus thynnus
Rest	7/2015	3	Tarragona	T1	No	R0715T11	No	menu	cooked	Thunnus albacares	Thunnus thynnus
Rest	04/09/2015	4	Girona	G4	No	R0915G41	No	menu	cooked	Thunnus albacares	Thunnus thynnus
Rest	29/10/2015	4	Girona	G3	No	R1015G31	No	menu	cooked	Thunnus albacares	Thunnus thynnus
Rest	12/2014	1	Barcelona	B2	No	R1214B21	Sí	asked	row	Thunnus thynnus	Thunnus thynnus
Rest	2/2015	2	Girona	G2	No	R0215G21	No	asked	row	Thunnus thynnus	Thunnus thynnus
Rest	2/2015	2	Girona	G4	No	R0215G41	No	asked	row	Thunnus thynnus	Thunnus thynnus
Rest	3/2015	2	Barcelona	B3	No	R0315B31	No	asked	row	Thunnus thynnus	Thunnus thynnus
Rest	3/2015	2	Barcelona	B4	No	R0315B41	No	asked	row	Thunnus thynnus	Thunnus thynnus
Rest	4/2015	2	Barcelona	B4	No	R0415B41	No	asked	row	Thunnus thynnus	Thunnus thynnus
Rest	4/2015	2	Tarragona	T4	No	R0415T41	No	asked	row	Thunnus thynnus	Thunnus thynnus
Rest	5/2015	3	Barcelona	B1	No	R0515B11	No	asked	row	Thunnus thynnus	Thunnus thynnus
Rest	5/2015	3	Barcelona	B2	No	R0515B21	No	asked	row	Thunnus thynnus	Thunnus thynnus
Rest	5/2015	3	Barcelona	B4	No	R0515B42	No	asked	row	Thunnus thynnus	Thunnus thynnus
Rest	5/2015	3	Girona	G2	No	R0515G21	No	asked	row	Thunnus thynnus	Thunnus thynnus
Rest	6/2015	3	Barcelona	B4	No	R0615B41	Sí	asked	row	Thunnus thynnus	Thunnus thynnus
Rest	7/2015	3	Girona	G1	No	R0715G11	No	asked	row	Thunnus thynnus	Thunnus thynnus
Rest	22/08/2015	4	Barcelona	B1	No	R0815B11	No	asked	row	Thunnus thynnus	Thunnus thynnus
Rest	1/2015	1	Barcelona	B2	No aplica	R0115B21	No aplica	asked	row	Thunnus albacares	Thunnus albacares
Rest	1/2015	1	Girona	G2	No aplica	R0115G21	No aplica	asked	row	Thunnus albacares	Thunnus albacares
Rest	2/2015	2	Tarragona	T3	No aplica	R0215T31	No aplica	asked	row	Thunnus albacares	Thunnus albacares
Rest	2/2015	2	Tarragona	T4	No aplica	R0215T42	No aplica	asked	row	Thunnus albacares	Thunnus albacares
Rest	4/2015	2	Tarragona	T2	No aplica	R0415T21	No aplica	asked	row	Thunnus albacares	Thunnus albacares
Rest	5/2015	3	Barcelona	B3	No aplica	R0515B31	No aplica	asked	row	Thunnus albacares	Thunnus albacares
Rest	5/2015	3	Barcelona	B4	No aplica	R0515B41	No aplica	asked	row	Thunnus albacares	Thunnus albacares
Rest	5/2015	3	Girona	G4	No aplica	R0515G41	No aplica	asked	row	Thunnus albacares	Thunnus albacares
Rest	7/2015	3	Tarragona	T4	No aplica	R0715T41	No aplica	asked	row	Thunnus albacares	Thunnus albacares
Rest	05/09/2015	4	Girona	G1	No aplica	R0915G11	No aplica	asked	row	Thunnus albacares	Thunnus albacares
Rest	18/09/2015	4	Barcelona	B4	No aplica	R0915B41	No aplica	asked	row	Thunnus albacares	Thunnus albacares
Rest	24/10/2015	4	Tarragona	T4	No aplica	R1015T41	No aplica	asked	row	Thunnus albacares	Thunnus albacares

Rest	31/10/2015	4	Girona	G2	No aplica	R1015G21	No aplica	asked	row	Thunnus albacares	Thunnus albacares
Rest	12/2014	1	Girona	G4	No	R1214G41	No	asked	row	Thunnus obesus	Thunnus thynnus
Rest	1/2015	1	Girona	G4	No	R0115G41	No	asked	row	Thunnus obesus	Thunnus thynnus
Rest	1/2015	1	Tarragona	T4	No	R0115T41	No	asked	row	Thunnus obesus	Thunnus thynnus
Rest	3/2015	2	Tarragona	T4	No	R0315T41	No	asked	row	Thunnus obesus	Thunnus thynnus
Rest	7/2015	3	Barcelona	B4	No	R0715B41	No	asked	row	Thunnus obesus	Thunnus thynnus
Rest	29/09/2015	4	Barcelona	B4	No	R0915B42	No	asked	row	Thunnus obesus	Thunnus thynnus
Rest	12/2014	1	Barcelona	B1	No	R1214B11	No	asked	row	Thunnus albacares	Thunnus thynnus
Rest	12/2014	1	Barcelona	B3	No	R1214B31	No	asked	row	Thunnus albacares	Thunnus thynnus
Rest	12/2014	1	Barcelona	B4	No	R1214B41	No	asked	row	Thunnus albacares	Thunnus thynnus
Rest	12/2014	1	Girona	G4	No	R1214G42	No	asked	row	Thunnus albacares	Thunnus thynnus
Rest	12/2014	1	Tarragona	T3	No	R1214T31	No	asked	row	Thunnus albacares	Thunnus thynnus
Rest	11/2014	1	Tarragona	T4	Sí	R1114T41	Sí	asked	row	Thunnus albacares	Thunnus thynnus
Rest	12/2014	1	Tarragona	T4	No	R1214T41	Sí	asked	row	Thunnus albacares	Thunnus thynnus
Rest	12/2014	1	Tarragona	T4	No	R1214T42	No	asked	row	Thunnus albacares	Thunnus thynnus
Rest	2/2015	2	Barcelona	B4	No	R0215B41	No	asked	row	Thunnus albacares	Thunnus thynnus
Rest	3/2015	2	Barcelona	B2	No	R0315B21	No	asked	row	Thunnus albacares	Thunnus thynnus
Rest	3/2015	2	Girona	G2	No	R0315G21	No	asked	row	Thunnus albacares	Thunnus thynnus
Rest	3/2015	2	Girona	G4	No	R0315G41	No	asked	row	Thunnus albacares	Thunnus thynnus
Rest	4/2015	2	Barcelona	B1	No	R0415B11	No	asked	row	Thunnus albacares	Thunnus thynnus
Rest	4/2015	2	Barcelona	B3	No	R0415B31	No	asked	row	Thunnus albacares	Thunnus thynnus
Rest	4/2015	2	Girona	G1	No	R0415G11	No	asked	row	Thunnus albacares	Thunnus thynnus
Rest	4/2015	2	Tarragona	T1	No	R0415T11	Sí	asked	row	Thunnus albacares	Thunnus thynnus
Rest	10/09/2015	4	Tarragona	T2	No	R0915T21	No	asked	row	Thunnus albacares	Thunnus thynnus
Rest	17/10/2015	4	Barcelona	B2	No	R1015B21	No	asked	row	Thunnus albacares	Thunnus thynnus
Rest	2/2015	2	Girona	G4	No aplica	R0215G42	No aplica	asked	row	Thunnus thynnus	Thunnus albacares
Rest	7/2015	3	Girona	G2	No aplica	R0615G21	No aplica	asked	row	Thunnus thynnus	Thunnus albacares
Rest	27/08/2015	4	Barcelona	B3	No aplica	R0815B31	No aplica	asked	row	Thunnus thynnus	Thunnus albacares
Rest	22/08/2015	4	Girona	G1	No aplica	R0815G11	No aplica	asked	row	Thunnus thynnus	Thunnus albacares
Rest	24/10/2015	4	Tarragona	T2	No aplica	R1015T21	No aplica	asked	row	Thunnus thynnus	Thunnus albacares
Rest	5/2015	3	Girona	G1	No aplica	R0515G11	No aplica	asked	row	Sarda sarda	Thunnus albacares
Rest	5/2015	3	Tarragona	T4	No aplica	R0515T42	No aplica	asked	row	Thunnus obesus	Thunnus albacares
Rest	6/2015	3	Barcelona	B2	No aplica	R0615B21	No aplica	asked	row	Thunnus obesus	Thunnus albacares
Rest	7/2015	3	Girona	G4	No aplica	R0715G41	No aplica	asked	row	Thunnus obesus	Thunnus albacares

Rest	09/09/2015	4	Tarragona	T4	No aplica	R0915T42	No aplica	asked	row	Thunnus obesus	Thunnus albacares
Rest	11/2014	1	Barcelona	B3	No	R1114B31	No	asked	row	Thunnus thynnus	- sin especificar -
Rest	2/2015	2	Tarragona	T4	No aplica	R0215T41	No aplica	asked	row	Thunnus thynnus	- sin especificar -
Rest	23/08/2015	4	Barcelona	B4	No aplica	R0815B41	No aplica	asked	row	Thunnus thynnus	- sin especificar -
Rest	22/08/2015	4	Barcelona	B2	No aplica	R0815B21	No aplica	asked	row	Thunnus thynnus	- sin especificar -
Rest	04/09/2015	4	Girona	G4	No aplica	R0915G42	No aplica	asked	row	Thunnus thynnus	- sin especificar -
Rest	1/2015	1	Tarragona	T2	No aplica	R0115T21	No aplica	asked	row	Thunnus obesus	- sin especificar -
Rest	6/2015	3	Girona	G4	No aplica	R0615G41	No aplica	asked	row	Thunnus obesus	- sin especificar -
Rest	11/2014	1	Barcelona	B2	No aplica	R1114B21	No aplica	asked	row	Thunnus albacares	- sin especificar -
Rest	11/2014	1	Girona	G4	No aplica	R1114G41	No aplica	asked	row	Thunnus albacares	- sin especificar -
Rest	11/2014	1	Tarragona	T2	No aplica	R1114T21	No aplica	asked	row	Thunnus albacares	- sin especificar -
Rest	2/2015	2	Barcelona	B2	No aplica	R0215B21	No aplica	asked	row	Thunnus albacares	- sin especificar -
Rest	2/2015	2	Tarragona	T2	No aplica	R0215T21	No aplica	asked	row	Thunnus albacares	- sin especificar -
Rest	3/2015	2	Tarragona	T3	No aplica	R0315T31	No aplica	asked	row	Thunnus albacares	- sin especificar -
Rest	6/2015	3	Tarragona	T2	No aplica	R0615T21	No aplica	asked	row	Thunnus albacares	- sin especificar -
Rest	6/2015	3	Tarragona	T4	No aplica	R0615T41	No aplica	asked	row	Thunnus albacares	- sin especificar -
Rest	7/2015	3	Tarragona	T2	No aplica	R0715T21	No aplica	asked	row	Thunnus albacares	- sin especificar -
Rest	22/08/2015	4	Girona	G2	No aplica	R0815G21	No aplica	asked	row	Thunnus albacares	- sin especificar -
Rest	17/10/2015	4	Barcelona	B3	No aplica	R1015B31	No aplica	asked	row	Thunnus albacares	- sin especificar -
Rest	12/2014	1	Girona	G2	No	R1214G21	No	menu	row	Thunnus thynnus	Thunnus thynnus
Rest	12/2014	1	Tarragona	T2	No	R1214T21	No	menu	row	Thunnus thynnus	Thunnus thynnus
Rest	1/2015	1	Tarragona	T3	No	R0115T31	Sí	menu	row	Thunnus thynnus	Thunnus thynnus
Rest	2/2015	2	Barcelona	B1	No	R0215B11	Sí	menu	row	Thunnus thynnus	Thunnus thynnus
Rest	2/2015	2	Girona	G3	No	R0215G31	No	menu	row	Thunnus thynnus	Thunnus thynnus
Rest	4/2015	2	Tarragona	T3	No	R0415T31	Sí	menu	row	Thunnus thynnus	Thunnus thynnus
Rest	6/2015	3	Girona	G3	No	R0615G31	No	menu	row	Thunnus thynnus	Thunnus thynnus
Rest	12/2014	1	Girona	G1	No	R1214G11	Sí	menu	row	Thunnus obesus	Thunnus thynnus
Rest	5/2015	3	Tarragona	T3	Sí	R0515T31	Sí	menu	row	Thunnus alalunga	Thunnus thynnus
Rest	29/08/2015	4	Girona	G3	No	R0815G31	Sí	menu	row	Thunnus albacares	Thunnus thynnus
